# Optimal location of subtrochanteric osteotomy in total hip arthroplasty for crowe type IV developmental dysplasia of hip

**DOI:** 10.1186/s12891-020-03248-8

**Published:** 2020-04-06

**Authors:** Zhe-Yu Huang, Hua Liu, Ming Li, Jing Ling, Jun-Hui Zhang, Zhi-Min Zeng

**Affiliations:** The Department of Orthopedics Surgery, Ningbo No. 6 Hospital, 1059# ZhongShan East Road, Ningbo, Zhejiang, 315040 People’s Republic of China

**Keywords:** Developmental dysplasia of the hip, Femoral shortening transverse osteotomy, Non-union, Contact area, Optimal location

## Abstract

**Background:**

When reconstructing a hip with developmental dysplasia and high dislocation, sub-trochanteric shortening osteotomy is typically needed for placing the acetabular component in the appropriate anatomical position. However, the procedure can result in complications such as non-union of the osteotomy. We evaluated the contact area and the coincidence rate between the proximal and distal fragments at different femoral osteotomy levels and lengths. We then determined the optimal location of subtrochanteric femoral shortening transverse osteotomy in patients with unilateral Crowe type IV developmental dysplasia of the hip (DDH). The consistency between the proximal and distal segments was assessed as a possible predictive indicator of the union at the osteotomy site.

**Methods:**

We retrospectively reviewed 57 patients with unilateral Crowe type IV DDH who underwent X-ray imaging of both hip joints. We labelled the inner and outer diameters of the circular ring as N (mm) and M (mm), respectively. We defined the overlapped area between the proximal and distal ring as contact area S (mm^2^), and the ratio of contact area to distal ring area as coincidence rate R.

**Results:**

N varied from 9.8–15.2 mm and M varied from 20.7–24 mm, both demonstrated a decreasing trend in the proximal to distal direction. At osteotomy lengths ranging from 0.5–2 cm, there were no differences in S between the different levels of osteotomy in each group. At osteotomy lengths ≤2.5 cm, a significant higher coincidence rate was noted from 2 cm below the lesser trochanter to other positions below the level. At osteotomy lengths from 3 to 5.5 cm, a significantly higher coincidence rate was observed from the level of 1.5 cm below the lesser trochanter to other positions below the level.

**Conclusions:**

Our findings suggest that femoral shortening transverse osteotomy at the optimal subtrochanteric level can predictably increase the contact area and coincidence rate, which may contribute to the union at the osteotomy site. Considering the stability of the prostheses, it appears appropriate that osteotomy location should be shifted slightly distally.

**Trial registration:**

Retrospectively registered.

## Background

Total hip arthroplasty (THA) is the gold standard treatment in end-stage hip disorders, such as osteoarthritis, rheumatoid arthritis, and hip dislocation that leads to significant pain and loss of function [1]. Performing THA in Crowe type IV developmental dysplasia of the hip (DDH) is technically demanding and does present many challenges to the surgeon in terms of both the femoral and acetabular sides. One of the most important steps is transferring the hip into the anatomical center of rotation for durable results and correcting the functions of the abductor muscles [2, 3]. When restoring the anatomical center of hip rotation, the leg may be lengthened by over 4 cm [4], which increases the risk of direct or indirect neurologic injury [5]. Consequently, subtrochanteric femoral shortening osteotomy was introduced to facilitate pulling down of the femur, correct the rotational abnormalities, preserve the proximal femoral metaphysis, and reduce the risk of nerve injury [6].

Various subtrochanteric osteotomy techniques with other cutting shapes, transverse, oblique, double-chevron, and Z-shaped, have been previously described [7–13]. Transverse osteotomy may be recommended because of the technical simplicity in adjusting the anteversion angle and minimal damage of the periosteum at the osteotomy site [14, 15]. However, the risk of non-union remains a major concern during the procedure [16, 17]. A limited bone contact area is a major disadvantage of transverse osteotomy, which may interfere with the bone healing process [18, 19]. The consistency of the interfaces and canal diameters between the proximal and distal segments may contribute to union; the two osteotomy interfaces should be as smooth as possible to maintain their intactness [20].

In order to assess the consistency between the proximal and distal segments as a potential predictive indicator of the union of the osteotomy site, we evaluated the contact area and coincidence rate between the proximal and distal fragments at different levels and lengths of femoral osteotomy. We then determined the optimal location of subtrochanteric femoral shortening transverse osteotomy in patients with Crowe type IV DDH.

## Methods

**Fig. 1:**
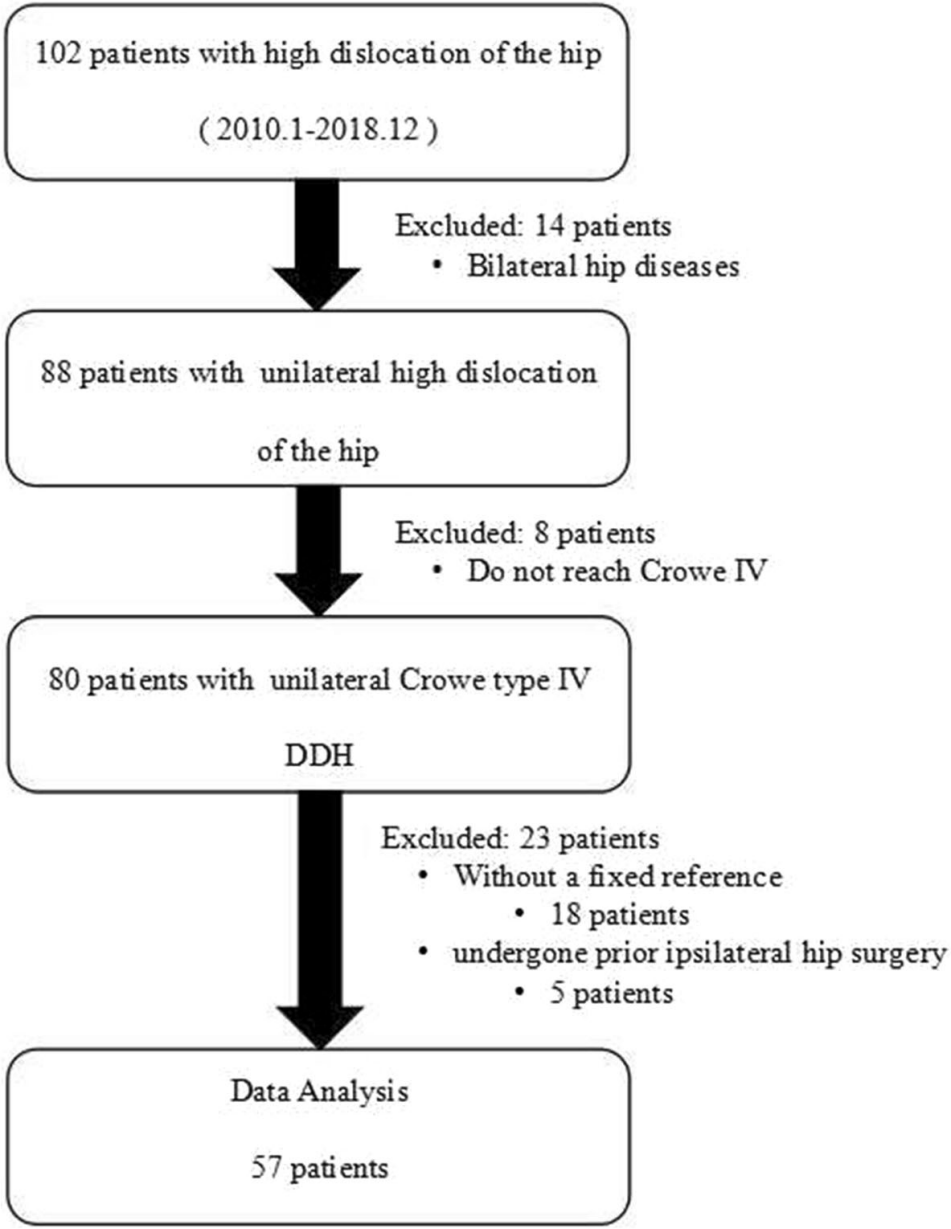
Flowchart depicting the selection of hips to be evaluated

We searched our Medical Image Database using our hospital’s picture archiving and communication system (PACS) and retrospectively reviewed the X-ray data of 102 patients with a high dislocation of the hip between 2010 and 2018. The inclusion criteria for this study were: (1) unilateral Crowe type IV DDH with a normal contralateral hip, and (2) the hip radiograph in the anteroposterior (AP) position included a fixed reference, such as a Yuan coin. The exclusion criteria included previous surgical history or fractures with deformities. Of the 102 patients, the following were excluded: 14 patients due to bilateral hip diseases, 8 patients without DDH of Crowe IV category, 18 patients without images with a fixed reference, and 5 patients who had undergone a prior ipsilateral hip surgery. Subsequently, we evaluated 57 patients with unilateral Crowe type IV DDH (Fig. 1), and these were comprised of 46 females and 11 males with an overall mean age of 53 years (range, 20–75).

**Fig. 2:**
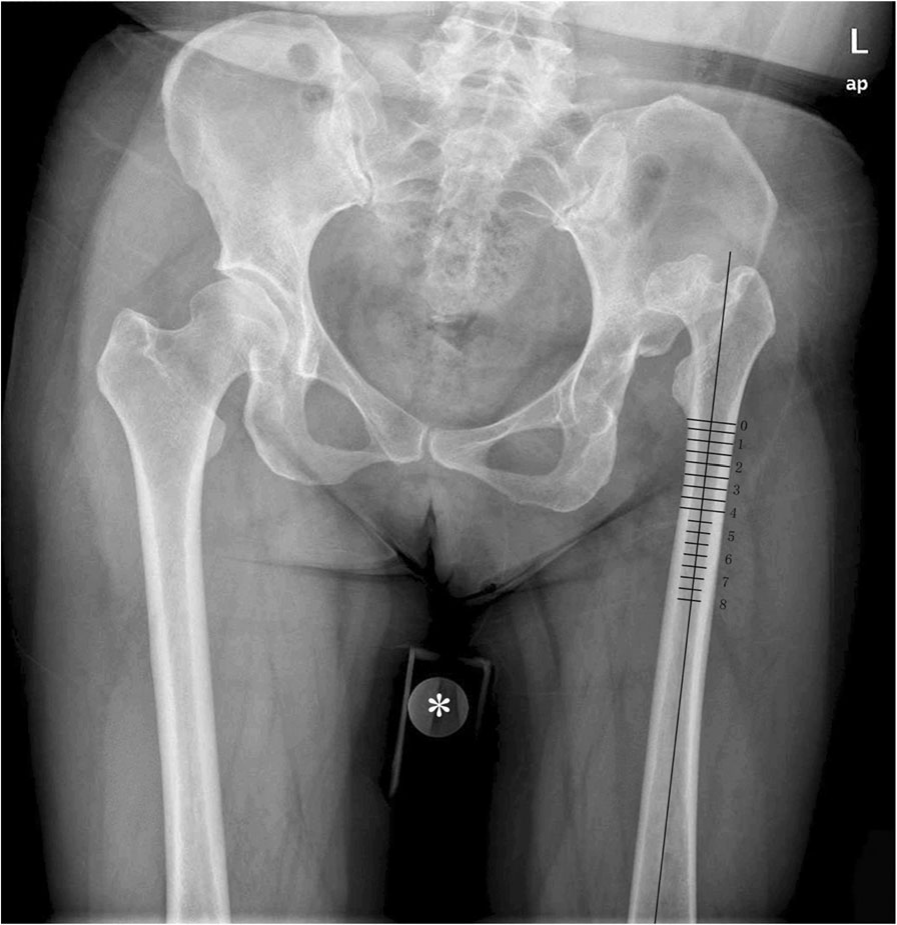
The inner and external diameters of each level of osteotomy on anteroposterior view on the radiograph. * A fixed reference, one-yuan coin

The AP radiographs were imported into Adobe Photoshop CS6 software v13.0 (Adobe Systems, Inc., San Jose, CA, USA) for image processing and calculating the measurements. First, we magnified or shrunk the radiographs to fit the actual size of the coin. Second, we drew two parallel lines along the inner and outer cortex of the femoral shaft, and then drew a midline of the two parallel lines as the femoral anatomic axis, and last, we drew sixteen vertical lines perpendicular to this axis from 0 to 8 cm below the lesser trochanter at 0.5 cm intervals (Fig. 2). The reasons we regarded the 8 cm mark below the lesser trochanter the most distant point were the following: (1) most osteotomies located at the proximal femur, including immediately distal to the lesser trochanter [21–25], are at 1–3 cm below the lesser trochanter [7, 20, 26, 27]; and (2) according to Su et al. [28], the femoral isthmus begins 8 cm below the lesser trochanter, which influences the stability of the prosthesis and, therefore, osteotomy should not be performed at or beyond this level.

**Fig. 3:**
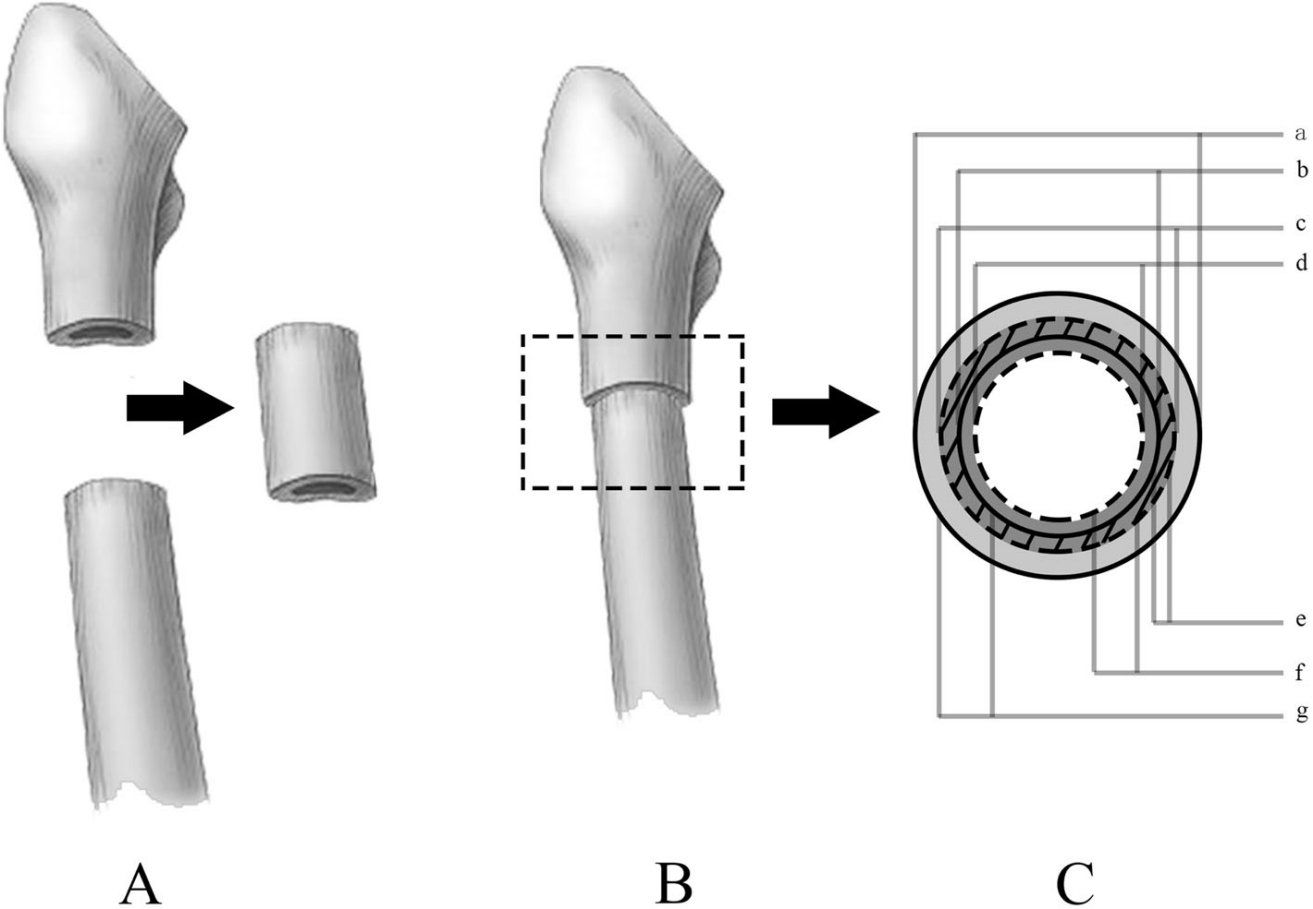
**A–C (A)** The segment is resected. **(B)** Coupling of the proximal and distal femurs. **(C)** Axis view of the interfaces: a: external diameter of the proximal segment (M_P_); b: inner diameter of the proximal segment (N_P_); c: external diameter of the distal segment (M_D_); d: inner diameter of the distal segment (N_D_); e: the overlapped area between the proximal and distal segments (contact area, S); f: area of the distal segment; and g: area of the proximal segment

In order to measure the area of cross-section of osteotomy, each section was assumed to be in the shape of a circular ring. We labelled the inner diameter of the circular ring as N (mm), and the external diameter as M (mm). We identified the overlapping area between the proximal and distal rings as the contact area, S (mm^2^), and the ratio of contact area to distal ring area as coincidence rate, R (Fig. 3). Mathematically, $S=\frac{\pi \left({M}_D^2-{N}_P^2\;\right)}{4}$, which was simplified to (M_D_^2^-N_P_^2^), and $R=\frac{M_D^2-{N}_P^2}{M_D^2-{N}_D^2}$. Overall, 17 sets of data for M and N, ranging from 0 to 8 cm below the lesser trochanter at 0.5 cm intervals were measured. We divided the length of osteotomy from 0.5 to 7 cm at intervals of 0.5 cm and named the proximal location of osteotomy as the level of osteotomy. According to the measured data, we obtained 14 groups of data for the contact area S and coincidence rate R for different osteotomy lengths. We only measured the levels from 0 to 8 cm below the lesser trochanter at intervals of 0.5 cm; therefore, different groups had a different number of levels of osteotomy, i.e., different groups had a different number of observations for S and R values. With an increase of 0.5 cm in the length of osteotomy, the number of observations decreased by one. Therefore, the group with an osteotomy length of 0.5 cm (0.5 L group) had 16 levels, the group with osteotomy at 1 cm length (1 L group) had 15 observations, and the group with osteotomy at 1.5 cm length (1.5 L group) had 14 observations, and so on. We incorporated a simple correction to the data. If M_P_ ≥ M_D_, and N_P_ ≤ N_D_, or M_D_ ≥ M_P_, and N_D_ ≤ N_P_, then R was equal to 1. If M_D_ ≤ N_P_, then coincidence rate R was equal to 0.

Statistical analyses were performed using SPSS software v24.0 (IBM Inc., Armonk, NY, USA). In each group, intra-group comparisons of S and R values were performed separately using one-way analysis of variance (ANOVA) and Student-Newman-Keuls test (q-test). The level of significance was set at *p* < 0.05.

## Results

The average N values were 15.2 and 9.8 mm at just below the lesser trochanter and 8 cm below the lesser trochanter, respectively. The average M values were 24 and 20.7 mm at approximately distal to the lesser trochanter and 8 cm below the lesser trochanter, respectively. Both, average M and N values, demonstrated a gradually decreasing trend distally. The detailed measurements are summarized in Table 1.

**Table 1:** The mean inner and external diameters at different levels of osteotomy

Level (cm)	Inner Diameter (mm)	External Diameter (mm)
0	15.2	24.0
0.5	13.8	23.3
1	12.8	22.7
1.5	12.1	22.3
2	11.5	21.9
2.5	11.1	21.5
3	11.0	21.3
3.5	10.8	21.1
4	10.7	20.9
4.5	10.7	20.9
5	10.6	20.8
5.5	10.4	20.8
6	10.3	20.7
6.5	10.2	20.7
7	10.0	20.7
7.5	9.9	20.7
8	9.8	20.7

In the 0.5 L group, the minimum S, 315.4 mm^2^, was at the level just below the lesser trochanter, while the maximum S, 339.9 mm^2^, was located at 1 cm below the lesser trochanter. There was no statistical difference in S between the different levels of the 0.5 L group (*p* = 1). In groups with osteotomy at 1–2 cm, no statistical difference in S between the levels of osteotomy in the three groups was observed (*p* = 0.807 for 1 L group, *p* = 0.36 for 1.5 L group, *p* = 0.071 for 2 L group). In the 2.5 L group, the minimum S, 231.4 mm^2^, appeared at the level of just below the lesser trochanter; the S value of this level was significantly lower than those in the other levels (*p* = 0.003). In the remaining nine groups, no statistical difference was noted in the levels of osteotomy, except for the level of just below the lesser trochanter, which had the smallest S value in each group. These data are summarized in Table 2; the results of ANOVA and q-test of each group are shown in Additional files 1, 2, 3, 4, 5, 6, 7, 8, 9, 10, 11, 12, 13 and 14.

**Table 2:** Contact Area at different levels and lengths of femoral osteotomy

Level (cm)	Length of Osteotomy (cm)													
	0.5	1	1.5	2	2.5	3	3.5	4	4.5	5	5.5	6	6.5	7
0	315.40	286.26	266.63	248.11	231.41	222.98	215.45	206.18	203.00	200.89	199.94	197.79	197.16	194.03
0.5	329.72	310.09	291.57	274.87	266.44	258.91	249.64	246.46	244.35	243.40	241.25	240.62	237.49	239.82
1	339.95	321.43	304.73	296.30	288.77	279.51	276.32	274.21	273.26	271.11	270.48	267.35	269.68	267.95
1.5	339.82	323.12	314.68	307.16	297.89	294.71	292.60	291.65	289.50	288.87	285.74	288.07	286.33	NA
2	337.05	328.62	321.09	311.83	308.64	306.54	305.59	303.43	302.81	299.68	302.01	300.27	NA	NA
2.5	337.96	330.43	321.16	317.98	315.87	314.92	312.77	312.14	309.01	311.34	309.61	NA	NA	NA
3	334.22	324.96	321.77	319.66	318.71	316.56	315.93	312.80	315.13	313.40	NA	NA	NA	NA
3.5	329.11	325.93	323.82	322.87	320.72	320.09	316.96	319.29	317.56	NA	NA	NA	NA	NA
4	329.62	327.52	326.57	324.41	323.78	320.66	322.99	321.25	NA	NA	NA	NA	NA	NA
4.5	327.73	326.77	324.62	323.99	320.87	323.20	321.46	NA	NA	NA	NA	NA	NA	NA
5	329.59	327.44	326.81	323.68	326.01	324.28	NA	NA	NA	NA	NA	NA	NA	NA
5.5	330.95	330.32	327.20	329.53	327.79	NA	NA	NA	NA	NA	NA	NA	NA	NA
6	332.95	329.82	332.15	330.42	NA	NA	NA	NA	NA	NA	NA	NA	NA	NA
6.5	331.94	334.27	332.54	NA	NA	NA	NA	NA	NA	NA	NA	NA	NA	NA
7	338.91	337.17	NA	NA	NA	NA	NA	NA	NA	NA	NA	NA	NA	NA
7.5	337.64	NA	NA	NA	NA	NA	NA	NA	NA	NA	NA	NA	NA	NA
8	NA	NA	NA	NA	NA	NA	NA	NA	NA	NA	NA	NA	NA	NA

*NA* Not Available

We also observed significant results in the R values. In the 0.5 L group, the minimum R, 86%, was observed at the level of just below the lesser trochanter. The R values were 90.8, 94.2, and 95.2% for the levels of 0.5, 1, and 1.5 cm below the lesser trochanter, respectively. A statistically significant decrease was found in R in the four proximal levels above the level of 2 cm below the lesser trochanter in comparison to those below this level (*p* < 0.0001). Similar results were observed in 1 L, 1.5 L, 2 L, and 2.5 L groups. In the 3 L group, the R values were 61, 73.5, and 81.5% for the levels of 0, 0.5, and 1 cm below the lesser trochanter, respectively. From the level of 1.5 cm below the lesser trochanter to other levels below this level, there was a significant increase in the R values in comparison to the three proximal levels. With respect to the length of osteotomy ranging from 3.5 to 5.5 cm, the results were similar to those of the 3 L group. In the 6 L group, the R values were 55.2, 67.5, 76.3, 82.4, and 86.3% for the levels of 0, 0.5, 1, 1.5, and 2 cm below the lesser trochanter, respectively. According to the q-test, there was a significant increase in the R value at the distal three levels in comparison to the two proximal levels. In the 6.5 L group, a significant increase was observed in the R value at the level of 1 and 1.5 cm below the lesser trochanter in comparison with the two proximal levels. Finally, in the 7 L group, no difference was found at the level of 0.5 and 1 cm below the lesser trochanter, except for the position of just below the lesser trochanter, which had the smallest R value. The results of the one-way ANOVA showed highly significant differences between the different levels in each group (*p* < 0.0001 for each group). These data are summarized in Table 3; the results of ANOVA and q-test of each group are showed in Additional files 1, 2, 3, 4, 5, 6, 7, 8, 9, 10, 11, 12, 13 and 14.

**Table 3:** Coincidence Rate at different levels and lengths of femoral osteotomy

Level (cm)	Length of Osteotomy (cm)													
	0.5	1	1.5	2	2.5	3	3.5	4	4.5	5	5.5	6	6.5	7
0	86.02%	77.04%	71.09%	66.59%	62.85%	61.06%	59.33%	57.31%	57.22%	56.49%	56.02%	55.24%	54.47%	53.96%
0.5	90.79%	84.98%	80.05%	76.61%	74.88%	73.47%	71.42%	71.27%	70.25%	69.24%	68.32%	67.54%	66.73%	66.94%
1	94.24%	89.60%	86.09%	84.56%	83.30%	81.50%	81.32%	80.30%	79.31%	78.11%	77.35%	76.33%	76.36%	75.58%
1.5	95.18%	91.74%	90.23%	89.06%	87.52%	87.40%	86.44%	85.30%	84.24%	83.51%	82.40%	82.38%	81.54%	NA
2	96.53%	95.01%	93.87%	92.44%	92.21%	91.40%	90.24%	89.20%	88.40%	87.30%	87.16%	86.27%	NA	NA
2.5	98.06%	96.80%	95.67%	95.28%	94.55%	93.36%	92.43%	91.66%	90.45%	90.31%	89.31%	NA	NA	NA
3	98.13%	96.89%	96.58%	95.81%	94.72%	93.81%	93.06%	91.74%	91.60%	90.68%	NA	NA	NA	NA
3.5	98.23%	97.84%	96.93%	95.88%	94.96%	94.09%	92.90%	92.69%	91.80%	NA	NA	NA	NA	NA
4	98.90%	98.06%	97.09%	96.16%	95.06%	94.03%	93.85%	92.99%	NA	NA	NA	NA	NA	NA
4.5	98.49%	97.31%	96.67%	95.54%	94.41%	94.22%	93.35%	NA	NA	NA	NA	NA	NA	NA
5	98.21%	97.41%	96.42%	95.09%	94.98%	94.98%	93.99%	NA	NA	NA	NA	NA	NA	NA
5.5	98.69%	97.86%	96.52%	96.20%	95.21%	NA	NA	NA	NA	NA	NA	NA	NA	NA
6	98.49%	97.35%	97.01%	96.20%	NA	NA	NA	NA	NA	NA	NA	NA	NA	NA
6.5	98.22%	97.86%	96.87%	NA	NA	NA	NA	NA	NA	NA	NA	NA	NA	NA
7	98.99%	98.16%	NA	NA	NA	NA	NA	NA	NA	NA	NA	NA	NA	NA
7.5	98.36%	NA	NA	NA	NA	NA	NA	NA	NA	NA	NA	NA	NA	NA
8	NA	NA	NA	NA	NA	NA	NA	NA	NA	NA	NA	NA	NA	NA

*NA* Not Available

## Discussion

In Crowe type IV DDH, the anatomy of the hip joint is markedly distorted due to acetabular and femoral bone stock deficiency, narrower femoral canal, excessive anteversion of the femur, valgus neck-shaft angle, posterior location of the greater trochanter, soft tissue contractures, lower limb length discrepancy, and dysfunctional hip abduction [29, 30]. With these abnormalities, it is difficult to achieve and maintain a reduction in the true acetabulum. Therefore, sub-trochanteric femoral shortening osteotomy was devised to make reduction easier; however, it can be associated with complications, such as limping, sciatic nerve injury, and non-union of the osteotomy, or compromise the long-term survival of the stem.

Non-union of osteotomy may lead to varus angulation, pain, loss of rotational stability, and prosthetic loosening [6, 7, 23]. The incidence of non-union at the osteotomy site ranges from 0 to 22% [18, 29, 31–33]. There are multiple contributing factors. The most important being the instability of the osteotomy site [34], which can be divided into three aspects. The first aspect is the type of the sub-trochanteric femoral shortening osteotomy; a transverse osteotomy was reported to have lower rotational stability than other techniques [6, 34], such as oblique, double-chevron, and Z-shaped. On the contrary, Muratli et al. [34] performed a biomechanical study comparing four different sub-trochanteric osteotomy groups and demonstrated that there was no single inherent feature that increased the stability of the osteotomy designs. A comprehensive meta-analysis by Li et al. [14] reported no significant difference between the transverse and modified methods in terms of complications and survival rates. Transverse osteotomy, however, is a simple technique to adjust the anteversion angle with minimal damage to the periosteum at the osteotomy site [14, 15]. Therefore, we chose this osteotomy technique as a viable mechanism for shortening the femur. The other two aspects that influence stability are the morphology of the femoral stem and the fixation method used. A cylindrical prosthesis, such as S-ROM stem (Courtesy of DePuy Orthopaedics, Warsaw, IN, USA), was recommended due to its distal slit and proximal fixation, which provides better distal engagement and facilitates rotational stability [25, 35, 36]. Several studies have also described transverse osteotomy with strut grafts using a cable or plate and screws for fixation [7, 32, 37]. Even cemented fixation of the stem has been used to improve the rotational stability [19, 22]. Other factors, which affect union, are the contact area between the proximal and distal segments [18–20], bone stock, and vascularity. However, a limited bone contact area is a major disadvantage of transverse osteotomy, which may interfere with the bone healing process [18, 19]. The consistency of the interfaces and canal diameters between the proximal and distal segments may contribute to union [20].

In order to promote union at the osteotomy site, we evaluated the contact area, S, and coincidence rate, R, between the proximal and distal fragments at different femoral osteotomy levels and lengths and then determined the optimal position of sub-trochanteric femoral shortening transverse osteotomy in patients with high developmental dysplasia.

Our results demonstrate that the dimensions of the femur, from 0 to 8 cm below the lesser trochanter, gradually decreased. However, the variations were less pronounced in comparison to the normal dimensions of the femur [38, 39]; the differences were 3.3 and 5.4 mm for the external and internal diameters, respectively. These results suggest that patients with Crowe type IV DDH have straighter intramedullary canals [30, 40]. Regarding the contact area and coincidence rate, different lengths of osteotomy presented different results. While the external and inner diameters of the cross-section of osteotomy did not change significantly in comparison to the normal dimensions of the femur, the coincidence rates were significantly higher at 2 cm below the lesser trochanter and below the level (0.5–2.5 L groups); at 1.5 cm below the lesser trochanter and below the level (3–5.5 L groups); at 1 cm below the lesser trochanter and below the level (6–6.5 L groups); and at 0.5 cm below the lesser trochanter and below the level (7 L group). Furthermore, with increasing size of the resected femoral bone fragment, the contact area and coincidence rate gradually decreased. For example, at levels of 2 cm below the lesser trochanter, the contact area and coincidence rate were 308.6 mm^2^ and 92.2% in the 2.5 L group, respectively; 306.5 mm^2^ and 91.4% in the 3 L group, respectively; and 305.6 mm^2^ and 90.2% in the 3.5 L group, respectively. These results indicate that the proximal femur varied greatly, in both size and shape, relative to the distal femur, and the distal femur had straighter intramedullary canals in patients with Crowe type IV DDH. The findings that the contact area was not significantly different between the levels in the 0.5 L–2 L groups except for the level immediately below the lesser trochanter with the smallest contact area in the 2.5 L–7 L groups, may be explained by the following two reasons: the dimensions of the proximal femur varied greatly relative to those of the distal femur, and the diameter of the proximal femur was greater than that of the distal femur. Consequently, it was better to perform the osteotomy at the distal femur. However, two aspects should be considered. First, the level of 8 cm below the lesser trochanter always reached the femoral isthmus according to a study of Su et al. [28], which may influence the stability of prosthesis; therefore, osteotomy should not be performed at or below this level. Second, the femoral stem should bridge the osteotomy site by a certain distance. Ozan et al. suggested that the femoral stem should pass from the osteotomy site for at least 4–5 cm [41], while Yang et al. believed that the femoral stem bridged the osteotomy site by at least 3 cm [42]; even a depth of at least 7 cm below the osteotomy site has been previously reported [22]. For primary arthroplasty, the size of the femoral stem is usually slightly more than 10 cm from the distal part of the lesser trochanter to the distal end of the stem; therefore, the osteotomy site should not be too distal, in order to allow the stem to bridge with sufficient length. Therefore, it seems appropriate that the location of osteotomy should be shifted slightly distally in consideration of these factors. For example, in the 7 L group, there were just three levels of osteotomy. The contact area and coincidence rate were 194 mm^2^ and 54%, respectively, at the level of just below the lesser trochanter, 239.8 mm^2^ and 66.9% at the level of 0.5 cm below the lesser trochanter, respectively, and 267.9 mm^2^ and 75.6% at the level of 1 cm below the lesser trochanter, respectively. Conclusively, it is better to choose the level of 1 cm below the lesser trochanter, although there were no significant differences between the levels at 1 and 0.5 cm below the lesser trochanter.

We recognize that there are several major limitations to our study. First, the number of patients was relatively small. However, the incidence of Crowe type IV DDH is relatively low, and it is difficult to obtain a larger data series from a single institution. Second, the method of assessing the radiographic diameter of the femoral canal was imprecise as the femoral canal is not precisely round; however, other studies [43–45] have used similar approximate methods to assess the diameter of the femoral canal, and, therefore, we believe our final assessments of the contact areas and coincidence rates are valid.

## Conclusions

Subtrochanteric femoral shortening transverse osteotomy is a suitable technique relative to THA in patients with high DDH. The optimal location of osteotomy varied according to the size of the resected femoral bone fragment. Our findings may provide a reference for surgeons performing shortening transverse osteotomy. Further studies are needed to identify the most suitable location of osteotomy and improve the union at the osteotomy site.

## Supplementary information

**Additional file 1:Table S1A** that shows the result of one-way ANOVA of 0.5 L group. B that shows the result of q-test of 0.5 L group for contact area. C that shows the q-test of q-test of 0.5 L group for coincidence rate.

**Additional file 2:Table S2A** that shows the result of one-way ANOVA of 1 L group. B that shows the result of q-test of 1 L group for contact area. C that shows the q-test of q-test of 1 L group for coincidence rate.

**Additional file 3:Table S3A** that shows the result of one-way ANOVA of 1.5 L group. B that shows the result of q-test of 1.5 L group for contact area. C that shows the q-test of q-test of 1.5 L group for coincidence rate.

**Additional file 4:Table S4A** that shows the result of one-way ANOVA of 2 L group. B that shows the result of q-test of 2 L group for contact area. C that shows the q-test of q-test of 2 L group for coincidence rate.

**Additional file 5:Table S5A** that shows the result of one-way ANOVA of 2.5 L group. 5B that shows the result of q-test of 2.5 L group for contact area. C that shows the q-test of q-test of 2.5 L group for coincidence rate.

**Additional file 6:Table S6A** that shows the result of one-way ANOVA of 3 L group. B that shows the result of q-test of 3 L group for contact area. C that shows the q-test of q-test of 3 L group for coincidence rate.

**Additional file 7:Table S7A** that shows the result of one-way ANOVA of 3.5 L group. B that shows the result of q-test of 3.5 L group for contact area. C that shows the q-test of q-test of 3.5 L group for coincidence rate.

**Additional file 8:Table S8A** that shows the result of one-way ANOVA of 4 L group. B that shows the result of q-test of 4 L group for contact area. C that shows the q-test of q-test of 4 L group for coincidence rate.

**Additional file 9:Table S9A** that shows the result of one-way ANOVA of 4.5 L group. B that shows the result of q-test of 4.5 L group for contact area. C that shows the q-test of q-test of 4.5 L group for coincidence rate.

**Additional file 10:Table S10A** that shows the result of one-way ANOVA of 5 L group. B that shows the result of q-test of 5 L group for contact area. C that shows the q-test of q-test of 5 L group for coincidence rate.

**Additional file 11:Table S11A** that shows the result of one-way ANOVA of 5.5 L group. B that shows the result of q-test of 5.5 L group for contact area. C that shows the q-test of q-test of 5.5 L group for coincidence rate.

**Additional file 12:Table S12A** that shows the result of one-way ANOVA of 6 L group. B that shows the result of q-test of 6 L group for contact area. C that shows the q-test of q-test of 6 L group for coincidence rate.

**Additional file 13:Table 13A** that shows the result of one-way ANOVA of 6.5 L group. B that shows the result of q-test of 6.5 L group for contact area. C that shows the q-test of q-test of 6.5 L group for coincidence rate.

**Additional file 14:Table S14A** that shows the result of one-way ANOVA of 7 L group. B that shows the result of q-test of 7 L group for contact area. C that shows the q-test of q-test of 7 L group for coincidence rate.

## Data Availability

The datasets used and/or analysed during the current study are available from the corresponding author on reasonable request.

## Abbreviations

DDH: Developmental dysplasia of the hip
THA: Total hip arthroplasty
PACS: Picture archiving and communication system
AP: Anteroposterior
ANOVA: Analysis of variance
q-test: Student-Newman-Keuls test
