# Supplementary material for: Optimal location of subtrochanteric osteotomy in total hip arthroplasty for crowe type IV developmental dysplasia of hip
Source: BMC Musculoskelet Disord. 2020 Apr 6;21:210. doi: 10.1186/s12891-020-03248-8 (PMC7137204; doi:10.1186/s12891-020-03248-8)
Supplement: Supplementary file 1 — Additional file 1:Table S1A that shows the result of one-way ANOVA of 0.5 L group. B that shows the result of q-test of 0.5 L group for contact area. C that shows the q-test of q-test of 0.5 L group for coincidence rate. [file 12891_2020_3248_MOESM1_ESM.doc]

|  | | Sum of Squares | df. | Mean Squares | F | Sig. |
| --- | --- | --- | --- | --- | --- | --- |
| Contact Area_0.5L | Inter-group | 33267.471 | 15 | 2217.831 | .118 | 1.000 |
|  | Intra-group | 16868767.850 | 896 | 18826.750 |  |  |
|  | Total | 16902035.320 | 911 |  |  |  |
| Coincidence Rate_0.5L | Inter-group | 1.090 | 15 | .073 | 32.356 | .000 |
|  | Intra-group | 2.012 | 896 | .002 |  |  |
|  | Total | 3.102 | 911 |  |  |  |

Table A1.1. One-way ANOVA of 0.5L group

Table A1.2. The q-test of 0.5L group for contact area

|  | | |
| --- | --- | --- |
|  | | |
| Level (cm) | N | Subset for Alpha = 0.05 |
|  |  | 1 |
| 0 | 57 | 315.4019 |
| 4.5 | 57 | 327.7254 |
| 3.5 | 57 | 329.1144 |
| 5 | 57 | 329.5905 |
| 4 | 57 | 329.6230 |
| 0.5 | 57 | 329.7209 |
| 5.5 | 57 | 330.9521 |
| 6.5 | 57 | 331.9404 |
| 6 | 57 | 332.9518 |
| 3 | 57 | 334.2221 |
| 2 | 57 | 337.0542 |
| 7.5 | 57 | 337.6430 |
| 2.5 | 57 | 337.9579 |
| 7 | 57 | 338.9096 |
| 1.5 | 57 | 339.8195 |
| 1 | 57 | 339.9477 |
| Sig. |  | 1.000 |

Table A1.3. The q-test of 0.5L group for coincidence rate

| Level (cm) | N | Subset for Alpha = 0.05 | | | | |
| --- | --- | --- | --- | --- | --- | --- |
|  |  | 1 | 2 | 3 | 4 | 5 |
| 0 | 57 | .86021 |  |  |  |  |
| 0.5 | 57 |  | .90787 |  |  |  |
| 1 | 57 |  |  | .94239 |  |  |
| 1.5 | 57 |  |  | .95182 | .95182 |  |
| 2 | 57 |  |  |  | .96534 | .96534 |
| 2.5 | 57 |  |  |  |  | .98057 |
| 3 | 57 |  |  |  |  | .98132 |
| 5 | 57 |  |  |  |  | .98211 |
| 6.5 | 57 |  |  |  |  | .98218 |
| 3.5 | 57 |  |  |  |  | .98230 |
| 7.5 | 57 |  |  |  |  | .98359 |
| 6 | 57 |  |  |  |  | .98485 |
| 4.5 | 57 |  |  |  |  | .98485 |
| 5.5 | 57 |  |  |  |  | .98689 |
| 4 | 57 |  |  |  |  | .98904 |
| 7 | 57 |  |  |  |  | .98989 |
| Sig. |  | 1.000 | 1.000 | .289 | .128 | .196 |
