# Supplementary material for: Optimal location of subtrochanteric osteotomy in total hip arthroplasty for crowe type IV developmental dysplasia of hip
Source: BMC Musculoskelet Disord. 2020 Apr 6;21:210. doi: 10.1186/s12891-020-03248-8 (PMC7137204; doi:10.1186/s12891-020-03248-8)
Supplement: Supplementary file 2 — Additional file 2:Table S2A that shows the result of one-way ANOVA of 1 L group. B that shows the result of q-test of 1 L group for contact area. C that shows the q-test of q-test of 1 L group for coincidence rate. [file 12891_2020_3248_MOESM2_ESM.doc]

|  | | Sum of Squares | df. | Mean Squares | F | Sig. |
| --- | --- | --- | --- | --- | --- | --- |
| Contact Area_1L | Inter-group | 118351.440 | 14 | 8453.674 | .451 | .957 |
|  | Intra-group | 15753333.750 | 840 | 18753.969 |  |  |
|  | Total | 15871685.190 | 854 |  |  |  |
| Coincidence Rate_1L | Inter-group | 2.974 | 14 | .212 | 38.482 | .000 |
|  | Intra-group | 4.638 | 840 | .006 |  |  |
|  | Total | 7.612 | 854 |  |  |  |

Table A2.1. One-way ANOVA of 1L group

Table A2.2. The q-test of 1L group for contact area

|  | | |
| --- | --- | --- |
|  | | |
| Level (cm) | N | Subset for Alpha = 0.05 |
|  |  | 1 |
| 0 | 57 | 286.2593 |
| 0.5 | 57 | 310.087 |
| 1 | 57 | 321.4332 |
| 1.5 | 57 | 323.1167 |
| 3 | 57 | 324.9568 |
| 3.5 | 57 | 325.9295 |
| 4.5 | 57 | 326.7749 |
| 5 | 57 | 327.437 |
| 4 | 57 | 327.5156 |
| 2 | 57 | 328.6223 |
| 6 | 57 | 329.823 |
| 5.5 | 57 | 330.3249 |
| 2.5 | 57 | 330.43 |
| 6.5 | 57 | 334.2704 |
| 7 | 57 | 337.1749 |
| Sig. |  | .807 |

Table A2.3. The q-test of 1L group for coincidence rate

| Level (cm) | N | Subset for Alpha = 0.05 | | | |  |
| --- | --- | --- | --- | --- | --- | --- |
|  |  | 1 | 2 | 3 | 4 | |
| 0 | 57 | 0.77041 |  |  |  | |
| 0.5 | 57 |  | 0.84982 |  |  | |
| 1 | 57 |  |  | 0.89604 |  | |
| 1.5 | 57 |  |  | 0.91736 |  | |
| 2 | 57 |  |  |  | 0.95012 | |
| 2.5 | 57 |  |  |  | 0.96799 | |
| 3 | 57 |  |  |  | 0.96893 | |
| 4.5 | 57 |  |  |  | 0.97313 | |
| 6 | 57 |  |  |  | 0.97349 | |
| 5 | 57 |  |  |  | 0.97406 | |
| 3.5 | 57 |  |  |  | 0.97843 | |
| 5.5 | 57 |  |  |  | 0.97864 | |
| 6.5 | 57 |  |  |  | 0.97864 | |
| 4 | 57 |  |  |  | 0.98057 | |
| 7 | 57 |  |  |  | 0.98164 | |
| Sig. |  | 1 | 1 | 0.126 | 0.459 | |
