# Supplementary material for: Optimal location of subtrochanteric osteotomy in total hip arthroplasty for crowe type IV developmental dysplasia of hip
Source: BMC Musculoskelet Disord. 2020 Apr 6;21:210. doi: 10.1186/s12891-020-03248-8 (PMC7137204; doi:10.1186/s12891-020-03248-8)
Supplement: Supplementary file 3 — Additional file 3:Table S3A that shows the result of one-way ANOVA of 1.5 L group. B that shows the result of q-test of 1.5 L group for contact area. C that shows the q-test of q-test of 1.5 L group for coincidence rate. [file 12891_2020_3248_MOESM3_ESM.doc]

|  | | Sum of Squares | df. | Mean Squares | F | Sig. |
| --- | --- | --- | --- | --- | --- | --- |
| Contact Area_1.5L | Inter-group | 243031.969 | 13 | 18694.767 | .999 | .450 |
|  | Intra-group | 14674147.810 | 784 | 18717.025 |  |  |
|  | Total | 14917179.780 | 797 |  |  |  |
| Coincidence Rate_1.5L | Inter-group | 4.651 | 13 | .358 | 39.329 | .000 |
|  | Intra-group | 7.132 | 784 | .009 |  |  |
|  | Total | 11.784 | 797 |  |  |  |

Table A3.1. One-way ANOVA of 1.5L group

Table A3.2. The q-test of 1.5L group for contact area

|  | | |
| --- | --- | --- |
|  | | |
| Level (cm) | N | Subset for Alpha = 0.05 |
|  |  | 1 |
| 0 | 57 | 266.6254 |
| 0.5 | 57 | 291.5725 |
| 1 | 57 | 304.7304 |
| 1.5 | 57 | 314.6847 |
| 2 | 57 | 321.0944 |
| 2.5 | 57 | 321.1647 |
| 3 | 57 | 321.7719 |
| 3.5 | 57 | 323.8221 |
| 4.5 | 57 | 324.6214 |
| 4 | 57 | 326.5651 |
| 5 | 57 | 326.8098 |
| 5.5 | 57 | 327.1961 |
| 6 | 57 | 332.153 |
| 6.5 | 57 | 332.5356 |
| Sig. |  | 0.36 |

Table A3.3. The q-test of 1.5L group for coincidence rate

| Level (cm) | N | Subset for Alpha = 0.05 | | | | |
| --- | --- | --- | --- | --- | --- | --- |
|  |  | 1 | 2 | 3 | 4 | 5 |
| 0 | 57 | 0.71092 |  |  |  |  |
| 0.5 | 57 |  | 0.80051 |  |  |  |
| 1 | 57 |  |  | 0.86088 |  |  |
| 1.5 | 57 |  |  |  | 0.9023 |  |
| 2 | 57 |  |  |  |  | 0.93869 |
| 2.5 | 57 |  |  |  |  | 0.95673 |
| 5 | 57 |  |  |  |  | 0.96415 |
| 5.5 | 57 |  |  |  |  | 0.96518 |
| 3 | 57 |  |  |  |  | 0.96584 |
| 4.5 | 57 |  |  |  |  | 0.96666 |
| 6.5 | 57 |  |  |  |  | 0.9687 |
| 3.5 | 57 |  |  |  |  | 0.96931 |
| 6 | 57 |  |  |  |  | 0.97006 |
| 4 | 57 |  |  |  |  | 0.97093 |
| Sig. |  | 1 | 1 | 1 | 1 | 0.732 |
