# Supplementary material for: Optimal location of subtrochanteric osteotomy in total hip arthroplasty for crowe type IV developmental dysplasia of hip
Source: BMC Musculoskelet Disord. 2020 Apr 6;21:210. doi: 10.1186/s12891-020-03248-8 (PMC7137204; doi:10.1186/s12891-020-03248-8)
Supplement: Supplementary file 4 — Additional file 4:Table S4A that shows the result of one-way ANOVA of 2 L group. B that shows the result of q-test of 2 L group for contact area. C that shows the q-test of q-test of 2 L group for coincidence rate. [file 12891_2020_3248_MOESM4_ESM.doc]

|  | | Sum of Squares | df. | Mean Squares | F | Sig. |
| --- | --- | --- | --- | --- | --- | --- |
| Contact Area_2L | Inter-group | 397583.743 | 12 | 33131.979 | 1.763 | .050 |
|  | Intra-group | 13684425.650 | 728 | 18797.288 |  |  |
|  | Total | 14082009.400 | 740 |  |  |  |
| Coincidence Rate_2L | Inter-group | 5.866 | 12 | .489 | 39.709 | .000 |
|  | Intra-group | 8.962 | 728 | .012 |  |  |
|  | Total | 14.827 | 740 |  |  |  |

Table A4.1. One-way ANOVA of 2L group

Table A4.2. The q-test of 2L group for contact area

|  | | |
| --- | --- | --- |
|  | | |
| Level (cm) | N | Subset for Alpha = 0.05 |
|  |  | 1 |
| 0 | 57 | 248.1109 |
| 0.5 | 57 | 274.8696 |
| 1 | 57 | 296.2984 |
| 1.5 | 57 | 307.1568 |
| 2 | 57 | 311.8291 |
| 2.5 | 57 | 317.9798 |
| 3 | 57 | 319.6646 |
| 3.5 | 57 | 322.8716 |
| 5 | 57 | 323.6811 |
| 4.5 | 57 | 323.9942 |
| 4 | 57 | 324.4116 |
| 5.5 | 57 | 329.5261 |
| 6 | 57 | 330.4182 |
| Sig. |  | 0.071 |

Table A4.3. The q-test of 2L group for coincidence rate

| Level (cm) | N | Subset for Alpha = 0.05 | | | | |
| --- | --- | --- | --- | --- | --- | --- |
|  |  | 1 | 2 | 3 | 4 | 5 |
| 0 | 57 | 0.66592 |  |  |  |  |
| 0.5 | 57 |  | 0.76607 |  |  |  |
| 1 | 57 |  |  | 0.84558 |  |  |
| 1.5 | 57 |  |  |  | 0.89058 |  |
| 2 | 57 |  |  |  | 0.92436 | 0.92436 |
| 5 | 57 |  |  |  |  | 0.95092 |
| 2.5 | 57 |  |  |  |  | 0.95284 |
| 4.5 | 57 |  |  |  |  | 0.95543 |
| 3 | 57 |  |  |  |  | 0.95805 |
| 3.5 | 57 |  |  |  |  | 0.95878 |
| 4 | 57 |  |  |  |  | 0.9616 |
| 5.5 | 57 |  |  |  |  | 0.96195 |
| 6 | 57 |  |  |  |  | 0.96199 |
| Sig. |  | 1 | 1 | 1 | 0.105 | 0.675 |
