# Supplementary material for: Optimal location of subtrochanteric osteotomy in total hip arthroplasty for crowe type IV developmental dysplasia of hip
Source: BMC Musculoskelet Disord. 2020 Apr 6;21:210. doi: 10.1186/s12891-020-03248-8 (PMC7137204; doi:10.1186/s12891-020-03248-8)
Supplement: Supplementary file 5 — Additional file 5:Table S5A that shows the result of one-way ANOVA of 2.5 L group. 5B that shows the result of q-test of 2.5 L group for contact area. C that shows the q-test of q-test of 2.5 L group for coincidence rate. [file 12891_2020_3248_MOESM5_ESM.doc]

|  | | Sum of Squares | df. | Mean Squares | F | Sig. |
| --- | --- | --- | --- | --- | --- | --- |
| Contact Area_2.5L | Inter-group | 532070.294 | 11 | 48370.027 | 2.572 | .003 |
|  | Intra-group | 12635883.660 | 672 | 18803.398 |  |  |
|  | Total | 13167953.960 | 683 |  |  |  |
| Coincidence Rate_2.5L | Inter-group | 6.650 | 11 | .605 | 38.350 | .000 |
|  | Intra-group | 10.594 | 672 | .016 |  |  |
|  | Total | 17.244 | 683 |  |  |  |

Table A5.1. One-way ANOVA of 2.5L group

Table A5.2. The q-test of 2.5L group for contact area

| Level (cm) | N | Subset for Alpha = 0.05 | |
| --- | --- | --- | --- |
|  |  | 1 | 2 |
| 0 | 57 | 231.4081 |  |
| 0.5 | 57 | 266.4377 | 266.4377 |
| 1 | 57 | 288.7705 | 288.7705 |
| 1.5 | 57 |  | 297.8916 |
| 2 | 57 |  | 308.6442 |
| 2.5 | 57 |  | 315.8725 |
| 3 | 57 |  | 318.714 |
| 3.5 | 57 |  | 320.7181 |
| 4.5 | 57 |  | 320.8654 |
| 4 | 57 |  | 323.7844 |
| 5 | 57 |  | 326.0111 |
| 5.5 | 57 |  | 327.7914 |
| Sig. |  | 0.066 | 0.375 |

Table A5.3. The q-test of 2.5L group for coincidence rate

| Level (cm) | N | Subset for Alpha = 0.05 | | | |  |
| --- | --- | --- | --- | --- | --- | --- |
|  |  | 1 | 2 | 3 | 4 | |
| 0 | 57 | 0.62847 |  |  |  | |
| 0.5 | 57 |  | 0.74881 |  |  | |
| 1 | 57 |  |  | 0.833 |  | |
| 1.5 | 57 |  |  | 0.87524 |  | |
| 2 | 57 |  |  |  | 0.92206 | |
| 4.5 | 57 |  |  |  | 0.94413 | |
| 2.5 | 57 |  |  |  | 0.94551 | |
| 3 | 57 |  |  |  | 0.94722 | |
| 3.5 | 57 |  |  |  | 0.94957 | |
| 5 | 57 |  |  |  | 0.94978 | |
| 4 | 57 |  |  |  | 0.95057 | |
| 5.5 | 57 |  |  |  | 0.95209 | |
| Sig. |  | 1 | 1 | 0.073 | 0.907 | |
