# Supplementary material for: Optimal location of subtrochanteric osteotomy in total hip arthroplasty for crowe type IV developmental dysplasia of hip
Source: BMC Musculoskelet Disord. 2020 Apr 6;21:210. doi: 10.1186/s12891-020-03248-8 (PMC7137204; doi:10.1186/s12891-020-03248-8)
Supplement: Supplementary file 6 — Additional file 6:Table S6A that shows the result of one-way ANOVA of 3 L group. B that shows the result of q-test of 3 L group for contact area. C that shows the q-test of q-test of 3 L group for coincidence rate. [file 12891_2020_3248_MOESM6_ESM.doc]

|  | | Sum of Squares | df. | Mean Squares | F | Sig. |
| --- | --- | --- | --- | --- | --- | --- |
| Contact Area_3L | Inter-group | 600670.278 | 10 | 60067.028 | 3.171 | .001 |
|  | Intra-group | 11667170.500 | 616 | 18940.212 |  |  |
|  | Total | 12267840.780 | 626 |  |  |  |
| Coincidence Rate_3L | Inter-group | 6.799 | 10 | .680 | 35.291 | .000 |
|  | Intra-group | 11.868 | 616 | .019 |  |  |
|  | Total | 18.668 | 626 |  |  |  |

Table A6.1. One-way ANOVA of 3L group

Table A6.2. The q-test of 3L group for contact area

| Level (cm) | N | Subset for Alpha = 0.05 | |
| --- | --- | --- | --- |
|  |  | 1 | 2 |
| 0 | 57 | 222.9761 |  |
| 0.5 | 57 | 258.9098 | 258.9098 |
| 1 | 57 | 279.5053 | 279.5053 |
| 1.5 | 57 |  | 294.7067 |
| 2 | 57 |  | 306.5368 |
| 2.5 | 57 |  | 314.9219 |
| 3 | 57 |  | 316.5605 |
| 3.5 | 57 |  | 320.0909 |
| 4 | 57 |  | 320.6556 |
| 4.5 | 57 |  | 323.1954 |
| 5 | 57 |  | 324.2763 |
| Sig. |  | 0.073 | 0.252 |

Table A6.3. The q-test of 3L group for coincidence rate

| Level (cm) | N | Subset for Alpha = 0.05 | | | | |
| --- | --- | --- | --- | --- | --- | --- |
|  |  | 1 | 2 | 3 | 4 |  |
| 0 | 57 | 0.61064 |  |  |  |  |
| 0.5 | 57 |  | 0.73468 |  |  |  |
| 1 | 57 |  |  | 0.81497 |  |  |
| 1.5 | 57 |  |  |  | 0.87404 |  |
| 2 | 57 |  |  |  | 0.91395 |  |
| 2.5 | 57 |  |  |  | 0.93363 |  |
| 3 | 57 |  |  |  | 0.93805 |  |
| 5 | 57 |  |  |  | 0.93991 |  |
| 4 | 57 |  |  |  | 0.94026 |  |
| 3.5 | 57 |  |  |  | 0.94093 |  |
| 4.5 | 57 |  |  |  | 0.94224 |  |
| Sig. |  | 1 | 1 | 1 | 0.15 |  |
