# Supplementary material for: Optimal location of subtrochanteric osteotomy in total hip arthroplasty for crowe type IV developmental dysplasia of hip
Source: BMC Musculoskelet Disord. 2020 Apr 6;21:210. doi: 10.1186/s12891-020-03248-8 (PMC7137204; doi:10.1186/s12891-020-03248-8)
Supplement: Supplementary file 7 — Additional file 7:Table S7A that shows the result of one-way ANOVA of 3.5 L group. B that shows the result of q-test of 3.5 L group for contact area. C that shows the q-test of q-test of 3.5 L group for coincidence rate. [file 12891_2020_3248_MOESM7_ESM.doc]

|  | | Sum of Squares | df. | Mean Squares | F | Sig. |
| --- | --- | --- | --- | --- | --- | --- |
| Contact Area_3.5L | Inter-group | 657255.949 | 9 | 73028.439 | 3.806 | .000 |
|  | Intra-group | 10744990.210 | 560 | 19187.483 |  |  |
|  | Total | 11402246.160 | 569 |  |  |  |
| Coincidence Rate_3.5L | Inter-group | 6.927 | 9 | .770 | 33.001 | .000 |
|  | Intra-group | 13.061 | 560 | .023 |  |  |
|  | Total | 19.987 | 569 |  |  |  |

Table A7.1. One-way ANOVA of 3.5L group

Table A7.2. The q-test of 3.5L group for contact area

| Level (cm) | N | Subset for Alpha = 0.05 | |
| --- | --- | --- | --- |
|  |  | 1 | 2 |
| 0 | 57 | 215.4482 |  |
| 0.5 | 57 | 249.6446 | 249.6446 |
| 1 | 57 | 276.3204 | 276.3204 |
| 1.5 | 57 |  | 292.5993 |
| 2 | 57 |  | 305.5863 |
| 2.5 | 57 |  | 312.7684 |
| 3 | 57 |  | 315.9333 |
| 3.5 | 57 |  | 316.9621 |
| 4.5 | 57 |  | 321.4607 |
| 4 | 57 |  | 322.9856 |
| Sig. |  | 0.05 | 0.11 |

Table A7.3. The q-test of 3.5L group for coincidence rate

| Level (cm) | N | Subset for Alpha = 0.05 | | | | |
| --- | --- | --- | --- | --- | --- | --- |
|  |  | 1 | 2 | 3 | 4 |  |
| 0 | 57 | 0.59325 |  |  |  |  |
| 0.5 | 57 |  | 0.7142 |  |  |  |
| 1 | 57 |  |  | 0.8132 |  |  |
| 1.5 | 57 |  |  | 0.86443 | 0.86443 |  |
| 2 | 57 |  |  |  | 0.90239 |  |
| 2.5 | 57 |  |  |  | 0.92425 |  |
| 3.5 | 57 |  |  |  | 0.92898 |  |
| 3 | 57 |  |  |  | 0.93056 |  |
| 4.5 | 57 |  |  |  | 0.93345 |  |
| 4 | 57 |  |  |  | 0.93852 |  |
| Sig. |  | 1 | 1 | 0.074 | 0.131 |  |
