# Supplementary material for: Optimal location of subtrochanteric osteotomy in total hip arthroplasty for crowe type IV developmental dysplasia of hip
Source: BMC Musculoskelet Disord. 2020 Apr 6;21:210. doi: 10.1186/s12891-020-03248-8 (PMC7137204; doi:10.1186/s12891-020-03248-8)
Supplement: Supplementary file 8 — Additional file 8:Table S8A that shows the result of one-way ANOVA of 4 L group. B that shows the result of q-test of 4 L group for contact area. C that shows the q-test of q-test of 4 L group for coincidence rate. [file 12891_2020_3248_MOESM8_ESM.doc]

|  | | Sum of Squares | df. | Mean Squares | F | Sig. |
| --- | --- | --- | --- | --- | --- | --- |
| Contact Area_4L | Inter-group | 692079.706 | 8 | 86509.963 | 4.459 | .000 |
|  | Intra-group | 9778135.396 | 504 | 19401.062 |  |  |
|  | Total | 10470215.100 | 512 |  |  |  |
| Coincidence Rate_4L | Inter-group | 6.786 | 8 | .848 | 30.696 | .000 |
|  | Intra-group | 13.927 | 504 | .028 |  |  |
|  | Total | 20.712 | 512 |  |  |  |

Table A8.1. One-way ANOVA of 4L group

Table A8.2. The q-test of 4L group for contact area

| Level (cm) | N | Subset for Alpha = 0.05 | |
| --- | --- | --- | --- |
|  |  | 1 | 2 |
| 0 | 57 | 206.183 |  |
| 0.5 | 57 | 246.4596 | 246.4596 |
| 1 | 57 |  | 274.213 |
| 1.5 | 57 |  | 291.6488 |
| 2 | 57 |  | 303.4328 |
| 2.5 | 57 |  | 312.1412 |
| 3 | 57 |  | 312.8046 |
| 3.5 | 57 |  | 319.2921 |
| 4 | 57 |  | 321.2509 |
| Sig. |  | 0.123 | 0.082 |

Table A8.3. The q-test of 4L group for coincidence rate

| Level (cm) | N | Subset for Alpha = 0.05 | | | | |
| --- | --- | --- | --- | --- | --- | --- |
|  |  | 1 | 2 | 3 | 4 |  |
| 0 | 57 | 0.5731 |  |  |  |  |
| 0.5 | 57 |  | 0.71267 |  |  |  |
| 1 | 57 |  |  | 0.80302 |  |  |
| 1.5 | 57 |  |  | 0.85301 | 0.85301 |  |
| 2 | 57 |  |  |  | 0.89198 |  |
| 2.5 | 57 |  |  |  | 0.91657 |  |
| 3 | 57 |  |  |  | 0.91738 |  |
| 3.5 | 57 |  |  |  | 0.92694 |  |
| 4 | 57 |  |  |  | 0.92994 |  |
| Sig. |  | 1 | 1 | 0.109 | 0.135 |  |
