# Supplementary material for: Optimal location of subtrochanteric osteotomy in total hip arthroplasty for crowe type IV developmental dysplasia of hip
Source: BMC Musculoskelet Disord. 2020 Apr 6;21:210. doi: 10.1186/s12891-020-03248-8 (PMC7137204; doi:10.1186/s12891-020-03248-8)
Supplement: Supplementary file 9 — Additional file 9:Table S9A that shows the result of one-way ANOVA of 4.5 L group. B that shows the result of q-test of 4.5 L group for contact area. C that shows the q-test of q-test of 4.5 L group for coincidence rate. [file 12891_2020_3248_MOESM9_ESM.doc]

|  | | Sum of Squares | df. | Mean Squares | F | Sig. |
| --- | --- | --- | --- | --- | --- | --- |
| Contact Area_4.5L | Inter-group | 644995.617 | 7 | 92142.231 | 4.707 | .000 |
|  | Intra-group | 8769045.889 | 448 | 19573.763 |  |  |
|  | Total | 9414041.506 | 455 |  |  |  |
| Coincidence Rate_4.5L | Inter-group | 6.065 | 7 | .866 | 27.100 | .000 |
|  | Intra-group | 14.323 | 448 | .032 |  |  |
|  | Total | 20.388 | 455 |  |  |  |

Table A9.1. One-way ANOVA of 4.5L group

Table A9.2. The q-test of 4.5L group for contact area

| Level (cm) | N | Subset for Alpha = 0.05 | |
| --- | --- | --- | --- |
|  |  | 1 | 2 |
| 0 | 57 | 202.9981 |  |
| 0.5 | 57 | 244.3523 | 244.3523 |
| 1 | 57 |  | 273.2625 |
| 1.5 | 57 |  | 289.4953 |
| 2 | 57 |  | 302.8056 |
| 2.5 | 57 |  | 309.0125 |
| 3 | 57 |  | 315.1346 |
| 3.5 | 57 |  | 317.5574 |
| Sig. |  | 0.115 | 0.079 |

Table A9.3. The q-test of 4.5L group for coincidence rate

| Level (cm) | N | Subset for Alpha = 0.05 | | | | |
| --- | --- | --- | --- | --- | --- | --- |
|  |  | 1 | 2 | 3 | 4 |  |
| 0 | 57 | 0.57222 |  |  |  |  |
| 0.5 | 57 |  | 0.7025 |  |  |  |
| 1 | 57 |  |  | 0.79305 |  |  |
| 1.5 | 57 |  |  | 0.84243 | 0.84243 |  |
| 2 | 57 |  |  |  | 0.88402 |  |
| 2.5 | 57 |  |  |  | 0.90452 |  |
| 3 | 57 |  |  |  | 0.91598 |  |
| 3.5 | 57 |  |  |  | 0.91804 |  |
| Sig. |  | 1 | 1 | 0.141 | 0.161 |  |
