# Supplementary material for: Optimal location of subtrochanteric osteotomy in total hip arthroplasty for crowe type IV developmental dysplasia of hip
Source: BMC Musculoskelet Disord. 2020 Apr 6;21:210. doi: 10.1186/s12891-020-03248-8 (PMC7137204; doi:10.1186/s12891-020-03248-8)
Supplement: Supplementary file 10 — Additional file 10:Table S10A that shows the result of one-way ANOVA of 5 L group. B that shows the result of q-test of 5 L group for contact area. C that shows the q-test of q-test of 5 L group for coincidence rate. [file 12891_2020_3248_MOESM10_ESM.doc]

|  | | Sum of Squares | df. | Mean Squares | F | Sig. |
| --- | --- | --- | --- | --- | --- | --- |
| Contact Area_5L | Inter-group | 575724.190 | 6 | 95954.032 | 4.769 | .000 |
|  | Intra-group | 7887324.152 | 392 | 20120.725 |  |  |
|  | Total | 8463048.341 | 398 |  |  |  |
| Coincidence Rate_5L | Inter-group | 5.445 | 6 | .908 | 23.983 | .000 |
|  | Intra-group | 14.834 | 392 | .038 |  |  |
|  | Total | 20.279 | 398 |  |  |  |

Table A10.1. One-way ANOVA of 5L group

Table A10.2. The q-test of 5L group for contact area

| Level (cm) | N | Subset for Alpha = 0.05 | |
| --- | --- | --- | --- |
|  |  | 1 | 2 |
| 0 | 57 | 200.8907 |  |
| 0.5 | 57 | 243.4018 | 243.4018 |
| 1 | 57 |  | 271.1089 |
| 1.5 | 57 |  | 288.8681 |
| 2 | 57 |  | 299.6768 |
| 2.5 | 57 |  | 311.3425 |
| 3 | 57 |  | 313.3998 |
| Sig. |  | 0.11 | 0.092 |

Table A10.3. The q-test of 5L group for coincidence rate

| Level (cm) | N | Subset for Alpha = 0.05 | | | | |
| --- | --- | --- | --- | --- | --- | --- |
|  |  | 1 | 2 | 3 | 4 |  |
| 0 | 57 | 0.56486 |  |  |  |  |
| 0.5 | 57 |  | 0.6924 |  |  |  |
| 1 | 57 |  |  | 0.78106 |  |  |
| 1.5 | 57 |  |  | 0.8351 | 0.8351 |  |
| 2 | 57 |  |  |  | 0.87297 |  |
| 2.5 | 57 |  |  |  | 0.90307 |  |
| 3 | 57 |  |  |  | 0.90675 |  |
| Sig. |  | 1 | 1 | 0.139 | 0.203 |  |
