# Supplementary material for: Optimal location of subtrochanteric osteotomy in total hip arthroplasty for crowe type IV developmental dysplasia of hip
Source: BMC Musculoskelet Disord. 2020 Apr 6;21:210. doi: 10.1186/s12891-020-03248-8 (PMC7137204; doi:10.1186/s12891-020-03248-8)
Supplement: Supplementary file 11 — Additional file 11:Table S11A that shows the result of one-way ANOVA of 5.5 L group. B that shows the result of q-test of 5.5 L group for contact area. C that shows the q-test of q-test of 5.5 L group for coincidence rate. [file 12891_2020_3248_MOESM11_ESM.doc]

|  | | Sum of Squares | df. | Mean Squares | F | Sig. |
| --- | --- | --- | --- | --- | --- | --- |
| Contact Area_5.5L | Inter-group | 487699.452 | 5 | 97539.890 | 4.708 | .000 |
|  | Intra-group | 6960864.105 | 336 | 20716.857 |  |  |
|  | Total | 7448563.557 | 341 |  |  |  |
| Coincidence Rate_5.5L | Inter-group | 4.557 | 5 | .911 | 20.662 | .000 |
|  | Intra-group | 14.821 | 336 | .044 |  |  |
|  | Total | 19.378 | 341 |  |  |  |

Table A11.1. One-way ANOVA of 5.5L group

Table A11.2. The q-test of 5.5L group for contact area

| Level (cm) | N | Subset for Alpha = 0.05 | |
| --- | --- | --- | --- |
|  |  | 1 | 2 |
| 0 | 57 | 199.9402 |  |
| 0.5 | 57 | 241.2482 | 241.2482 |
| 1 | 57 |  | 270.4818 |
| 1.5 | 57 |  | 285.7393 |
| 2 | 57 |  | 302.0068 |
| 2.5 | 57 |  | 309.6077 |
| Sig. |  | 0.126 | 0.085 |

Table A11.3. The q-test of 5.5L group for coincidence rate

| Level (cm) | N | Subset for Alpha = 0.05 | | | | |
| --- | --- | --- | --- | --- | --- | --- |
|  |  | 1 | 2 | 3 | 4 |  |
| 0 | 57 | 0.56016 |  |  |  |  |
| 0.5 | 57 |  | 0.68322 |  |  |  |
| 1 | 57 |  |  | 0.7735 |  |  |
| 1.5 | 57 |  |  | 0.82403 | 0.82403 |  |
| 2 | 57 |  |  |  | 0.87159 |  |
| 2.5 | 57 |  |  |  | 0.89314 |  |
| Sig. |  | 1 | 1 | 0.2 | 0.186 |  |
