# Supplementary material for: Optimal location of subtrochanteric osteotomy in total hip arthroplasty for crowe type IV developmental dysplasia of hip
Source: BMC Musculoskelet Disord. 2020 Apr 6;21:210. doi: 10.1186/s12891-020-03248-8 (PMC7137204; doi:10.1186/s12891-020-03248-8)
Supplement: Supplementary file 12 — Additional file 12:Table S12A that shows the result of one-way ANOVA of 6 L group. B that shows the result of q-test of 6 L group for contact area. C that shows the q-test of q-test of 6 L group for coincidence rate. [file 12891_2020_3248_MOESM12_ESM.doc]

|  | | Sum of Squares | df. | Mean Squares | F | Sig. |
| --- | --- | --- | --- | --- | --- | --- |
| Contact Area_6L | Inter-group | 382064.145 | 4 | 95516.036 | 4.397 | .002 |
|  | Intra-group | 6082231.084 | 280 | 21722.254 |  |  |
|  | Total | 6464295.229 | 284 |  |  |  |
| Coincidence Rate_6L | Inter-group | 3.528 | 4 | .882 | 16.925 | .000 |
|  | Intra-group | 14.592 | 280 | .052 |  |  |
|  | Total | 18.121 | 284 |  |  |  |

Table A12.1. One-way ANOVA of 6L group

Table A12.2. The q-test of 6L group for contact area

| Level (cm) | N | Subset for Alpha = 0.05 | |
| --- | --- | --- | --- |
|  |  | 1 | 2 |
| 0 | 57 | 197.7867 |  |
| 0.5 | 57 | 240.6211 | 240.6211 |
| 1 | 57 |  | 267.3530 |
| 1.5 | 57 |  | 288.0693 |
| 2 | 57 |  | 300.2721 |
| Sig. |  | .122 | .137 |

Table A12.3. The q-test of 6L group for coincidence rate

| Level (cm) | N | Subset for Alpha = 0.05 | | |  |
| --- | --- | --- | --- | --- | --- |
|  |  | 1 | 2 | 3 | |
| 0 | 57 | .55238 |  |  | |
| 0.5 | 57 |  | .67539 |  | |
| 1 | 57 |  |  | .76325 | |
| 1.5 | 57 |  |  | .82379 | |
| 2 | 57 |  |  | .86273 | |
| Sig. |  | 1.000 | 1.000 | .054 | |
