# Supplementary material for: Optimal location of subtrochanteric osteotomy in total hip arthroplasty for crowe type IV developmental dysplasia of hip
Source: BMC Musculoskelet Disord. 2020 Apr 6;21:210. doi: 10.1186/s12891-020-03248-8 (PMC7137204; doi:10.1186/s12891-020-03248-8)
Supplement: Supplementary file 13 — Additional file 13:Table 13A that shows the result of one-way ANOVA of 6.5 L group. B that shows the result of q-test of 6.5 L group for contact area. C that shows the q-test of q-test of 6.5 L group for coincidence rate. [file 12891_2020_3248_MOESM13_ESM.doc]

|  | | Sum of Squares | df. | Mean Squares | F | Sig. |
| --- | --- | --- | --- | --- | --- | --- |
| Contact Area_6.5L | Inter-group | 264161.878 | 3 | 88053.959 | 3.794 | .011 |
|  | Intra-group | 5199351.198 | 224 | 23211.389 |  |  |
|  | Total | 5463513.076 | 227 |  |  |  |
| Coincidence Rate_6.5L | Inter-group | 2.424 | 3 | .808 | 12.806 | .000 |
|  | Intra-group | 14.134 | 224 | .063 |  |  |
|  | Total | 16.559 | 227 |  |  |  |

Table A13.1. One-way ANOVA of 6.5L group

Table A13.2. The q-test of 6.5L group for contact area

| Level (cm) | N | Subset for Alpha = 0.05 | |
| --- | --- | --- | --- |
|  |  | 1 | 2 |
| 0 | 57 | 197.1595 |  |
| 0.5 | 57 | 237.4923 | 237.4923 |
| 1 | 57 |  | 269.6830 |
| 1.5 | 57 |  | 286.3346 |
| Sig. |  | .159 | .203 |

Table A13.3. The q-test of 6.5L group for coincidence rate

| Level (cm) | N | Subset for Alpha = 0.05 | | | |
| --- | --- | --- | --- | --- | --- |
|  |  | 1 | 2 | 3 |  |
| 0 | 57 | .54468 |  |  |  |
| 0.5 | 57 |  | .66726 |  |  |
| 1 | 57 |  |  | .76358 |  |
| 1.5 | 57 |  |  | .81537 |  |
| Sig. |  | 1.000 | 1.000 | .272 |  |
