# Supplementary material for: Optimal location of subtrochanteric osteotomy in total hip arthroplasty for crowe type IV developmental dysplasia of hip
Source: BMC Musculoskelet Disord. 2020 Apr 6;21:210. doi: 10.1186/s12891-020-03248-8 (PMC7137204; doi:10.1186/s12891-020-03248-8)
Supplement: Supplementary file 14 — Additional file 14:Table S14A that shows the result of one-way ANOVA of 7 L group. B that shows the result of q-test of 7 L group for contact area. C that shows the q-test of q-test of 7 L group for coincidence rate. [file 12891_2020_3248_MOESM14_ESM.doc]

|  | | Sum of Squares | df. | Mean Squares | F | Sig. |
| --- | --- | --- | --- | --- | --- | --- |
| Contact Area_7L | Inter-group | 158683.096 | 2 | 79341.548 | 3.239 | .042 |
|  | Intra-group | 4114680.020 | 168 | 24492.143 |  |  |
|  | Total | 4273363.116 | 170 |  |  |  |
| Coincidence Rate_7L | Inter-group | 1.350 | 2 | .675 | 8.781 | .000 |
|  | Intra-group | 12.917 | 168 | .077 |  |  |
|  | Total | 14.268 | 170 |  |  |  |

Table A14.1. One-way ANOVA of 7L group

Table A14.2. The q-test of 7L group for contact area

| Level (cm) | N | Subset for Alpha = 0.05 | |
| --- | --- | --- | --- |
|  |  | 1 | 2 |
| 0 | 57 | 194.0307 |  |
| 0.5 | 57 | 239.8223 | 239.8223 |
| 1 | 57 |  | 267.9482 |
| Sig. |  | .120 | .339 |

Table A14.3. The q-test of 7L group for coincidence rate

| Level (cm) | N | Subset for Alpha = 0.05 | |
| --- | --- | --- | --- |
|  |  | 1 | 2 |
| 0 | 57 | .53957 |  |
| 0.5 | 57 |  | .66938 |
| 1 | 57 |  | .75580 |
| Sig. |  | 1.000 | .098 |
